# Swainsonine exposure induces impairment of host immune response in pregnant BALB/c mice

**DOI:** 10.1186/s12865-015-0114-z

**Published:** 2015-09-03

**Authors:** Yanchun Hu, Lei Wu, Chengmin Wang, Jing Luo, Fei Liao, Hui Tan, Hongxuan He

**Affiliations:** Institute of Zoology, Chinese Academy of Sciences, Beijing, 100101 P.R. China; Department of Clinical Veterinary Medicine, College of Veterinary Medicine, Sichuan Agricultural University, Wenjiang, 611130 P.R. China

## Abstract

**Background:**

Swainsonine can cause serious disorders in reproduction of livestock, affecting both corpora lutea and reproductive hormone. The purpose of this study was to investigate the mechanisms of swainsonine about the immunotoxic effects on pregnant mice in vivo.

**Results:**

The peripheral Th1/Th2 was detected by Ionomycin and phorbol myristate acetate (PMA)-stimulating peripheral blood mononuclear cells (PBMC) of phase pregnant mice. Relevant cytokines in serum was evaluated after exposed to different dose of swainsonine. Gene expression of IL-1β, IFN-γ, TNF-α, IL-4 and IL-10 in PBMC was assessed by real-time PCR. Swainsonine caused vacuolization phenomenon of lutein cells and a dose-effect relationship. The IL-1β, IFN-γ and TNF-α were promoted, but IL-4 and IL-10 were suppressed in serum. Swainsonine significantly increased IL-1β, IFN-γ and TNF-α nuclear translocation and decreased IL-4 and IL-10. Swainsonine resulted in a significant shift of peripheral Th1/Th2 paradigm to Th1.

**Conclusions:**

Our data demonstrate that the immunomodulatory of swainsonine disturbed the regular immunologic state of the pregnant mice. This may increase the risk of abortion and probably resulted in serious disorders in reproduction of livestock.

## Background

Swainsonine, one of the indolizidine alkaloid isolated from locoweeds (including *Astragalus* spp. and *Oxytropis* spp.) [1, 2], is the main toxin-resulting in animal locoism which clinical syndrome showed dispirited behavior, insensitivity, abortion, fertility problems, amniotic membrane swelling and even serious vacuolar degeneration of the nerves and the internal organs [3]. Swainsonine, whose structure is similar to mannose but has higher affinity with mannosidase than mannose, is a well-known inhibitor of lysosomal α-mannosidase and Golgi mannosidase II. Different cells, especially neurons and corpus luteum showed vacuolation, because swainsonine disturb mannose metabolism and the production of a mixture of mannose and asparagine polysaccharide [4, 5]. Swainsonine can inhibit essential enzymes in glycoprotein metabolism, resulting in long-term integration of hybrid sugars which disrupt the cellular metabolism, and can also cause disorders of hormone and enzyme synthesis and receptor binding [6]. Ingestion of swainsonine-containing plants can decrease serum progesterone concentration which is essential to early embryo development, implantation and maintenance of pregnancy [7–10]. Vacuolation of corpus luteum cells could be observed in pregnant and just aborted animals after long time ingestion of swainsonine-containing plants [11, 12], but it is not clearly if vacuolation is the major cause of the abortions observed following locoweed ingestion, or the disorder of reproductive hormone [3].

T-helper (Th) cells, as a part of pregnancy immunology, play an important role in modulating immune responses to ensure a successful pregnancy and fetus development [13–15]. Fetus is a source of alloantigens to maternal immunity during pregnancy, avoiding Th1-type immunity attack [16]. However, a lot of recurrent spontaneous abortion and preeclampsia may happen if predominant Th1-type immunity occur [17–19]. The decrease of Th1 and increase of Th2 are necessary to protect the fetus without compromising the mother, as Th1 produces an array of inflammatory cytokines (including IFN-γ, IL-2) and Th2 produces anti-inflammatory cytokines (IL-4) and IL-10 [13, 20].

Swainsonine as an immunomodulator agent, can enhance phagocytosis activity and hydrogen peroxide production by macrophages and lead to elevated secretion of certain glycoproteins and inflammatory cytokines including interferon-γ, and also have efficient function to induce progenitor cell proliferation, release into the circulation in rodents and have been considered to boost immune cell function in cancer patients [21–24]. These suggest that swainsonine may disrupt the pregnancy immunology by promoting autoimmunity or increasing Th1 immunity and suppressing Th2 immunity and even leading to abortion. To explore this possibility, pregnant mice were exposed to swainsonine orally, then the key cytokines of pregnancy immunology in serum and their gene expression were examined.

## Results

### Pregnancy status

All mice in the experiment were pregnant initially and had a normal state of appetite, drinking and faeces. We did euthanasia and separated the uterus and ovary for more analysis. The body weight of the mice treat with 3.2 mg•kg^−1^•d^−1^ dropped more than 15 % suddenly in 16th ~ 18th day of the pregnancy, and the aborted fetuses were found in the cages. We considered the mice were abortion, and did not do any other analysis on them.

### Effects of different concentrations of swainsonine on lutein cell

**Fig. 1:**
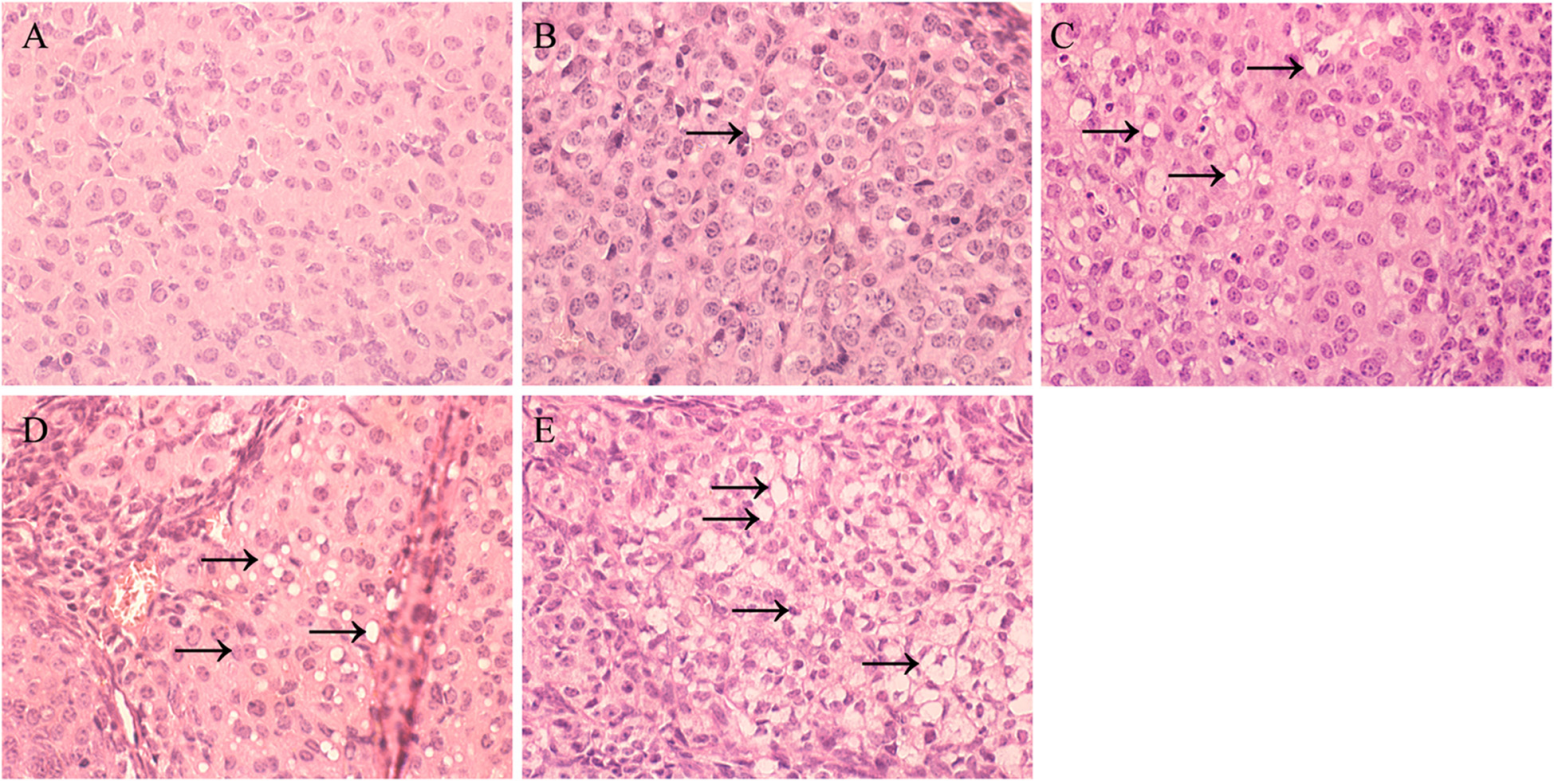
Histopathological slide of the corpus luteum. From the histopathological studies, vacuolation were observed after swainsonine treatment (**b, c, d, e**) but not for control group (**a**), as indicated by the arrows. The dose of 0.2 and 0.4 mg•kg^−1^•d^−1^ induced relatively vacuolation (**b, c**), but the group that received the dose of 0.8 and 1.6 mg•kg^−1^•d^−1^ showed serious cellular damage evidenced by vacuolation

In experiments to assess whether it is feasible to expose the experimental animals to swainsonine by orally route or not, histopathology of corpus luteum were analyzed. Microscopically, corpus luteum tissue exposed to different concentrations of swainsonine showed different degrees of degenerative changes when compared to the control group. On an average, degenerative changes noted with the treatment with swainsonine were almost the same but differed in intensity of lesions and cellular degeneration and death. However, vacuolation of the luteum cell (the classical histologic presentation of locoism) was seen at the dose of 0.4 mg•kg^−1^•d^−1^ and higher regularly or at the dose of 0.2 mg•kg^−1^•d^−1^ occasionally. High dose groups (0.8 and 1.6 mg•kg^−1^•d^−1^) showed serious cellular vacuolation (Fig. 1).

### Th1 and Th2 cytokine concentration between five groups at baseline

**Fig. 2:**
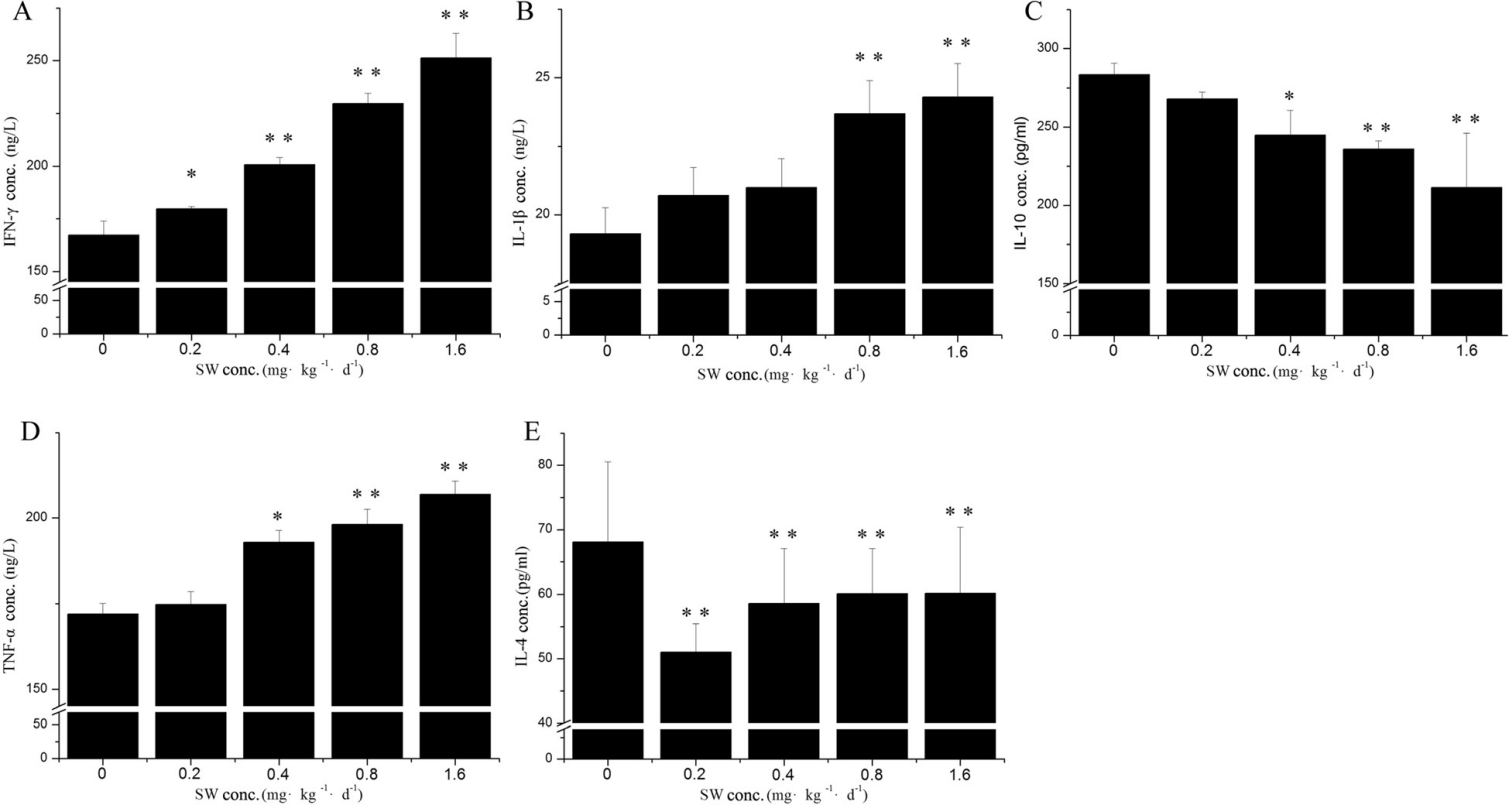
Th1 and Th2 cytokines concentration among the four groups and control (0 mg•kg-1•d-1) at baseline. Proinflammatory cytokines like TNF-α and IL-1β, and Th1 cytokine IFN-γ were significantly increased (*P* < 0.05) in cases of mice exposed to swainsonine, compared with the control group in a dose-related manner. **a**, **d**, **e** IL-1β and TNF-αincreased very significantly (*P* < 0.01) in peripheral blood leukocytes when exposed to swainsonine at the dose of 0.8 mg•kg^−1^•d^−1^ or higher dose (**b**, **c**). But for IFN-γ, a significant (*P* < 0.05) increase was observed with the treatment at a dose of 0.2 mg•kg^−1^•d^−1^. IL-4, as the Th2 cytokines and IL-10 were lower in experimental groups than in control group (*P* < 0.05), also in a dose-related manner. Only the dose of 0.2 mg•kg^−1^•d^−1^(*P* < 0.05) for IL-4 and the dose of 0.4 mg•kg^−1^•d-1 (*P* < 0.05) or higher dose (*P* < 0.01) for IL-10 showed inhibitory effect

To further examine the effect of different concentration of swainsonine in the inhibition of cytokine production, we examined the expression of cytokine in the peripheral blood by ELISA. The distribution of various cytokines among cases and control has been summarized in Fig. 2. Proinflammatory cytokines such as TNF-α and IL-1β, and the Th1 signature cytokine IFN-γ were significantly increased (*p* < 0.05) in mice exposed to mice exposed to swainsonine, compared with control group in a dose-related manner (Fig. 2a, d, e). IL-1β and TNF-α was significantly increased (*P* < 0.01) in peripheral blood leukocytes when swainsonine exposed at the dose of 0.8 mg•kg^−1^•d^−1^ or higher dose (Fig. 2b, c). Moreover, for IFN-γ, a significant (*P* < 0.05) increasing was observed with the treatment of a dose of 0.2 mg•kg^−1^•d^−1^, and significantly (*P* < 0.01) decreasing IL-4 and IL-10 were lower in experiment groups than in control (*P* < 0.05). Only in the dose of 0.2 mg•kg^−1^•d^−1^ (*P* < 0.05) or higher dose (*P* < 0.01) for IL-4 and in the dose of 0.4 mg•kg^−1^•d^−1^ (*P* < 0.05) or higher dose (*P* < 0.01) for IL-10 showed inhibitory effect. The results suggested that swainsonine administered orally for 13 d promoted Th1 cytokines and inhibited Th2 cytokines in serum of pregnant mice.

### Swainsonine stimulates IL-1β, IFN-γ, TNF-α and suppresses IL-4, IL-10

**Fig. 3:**
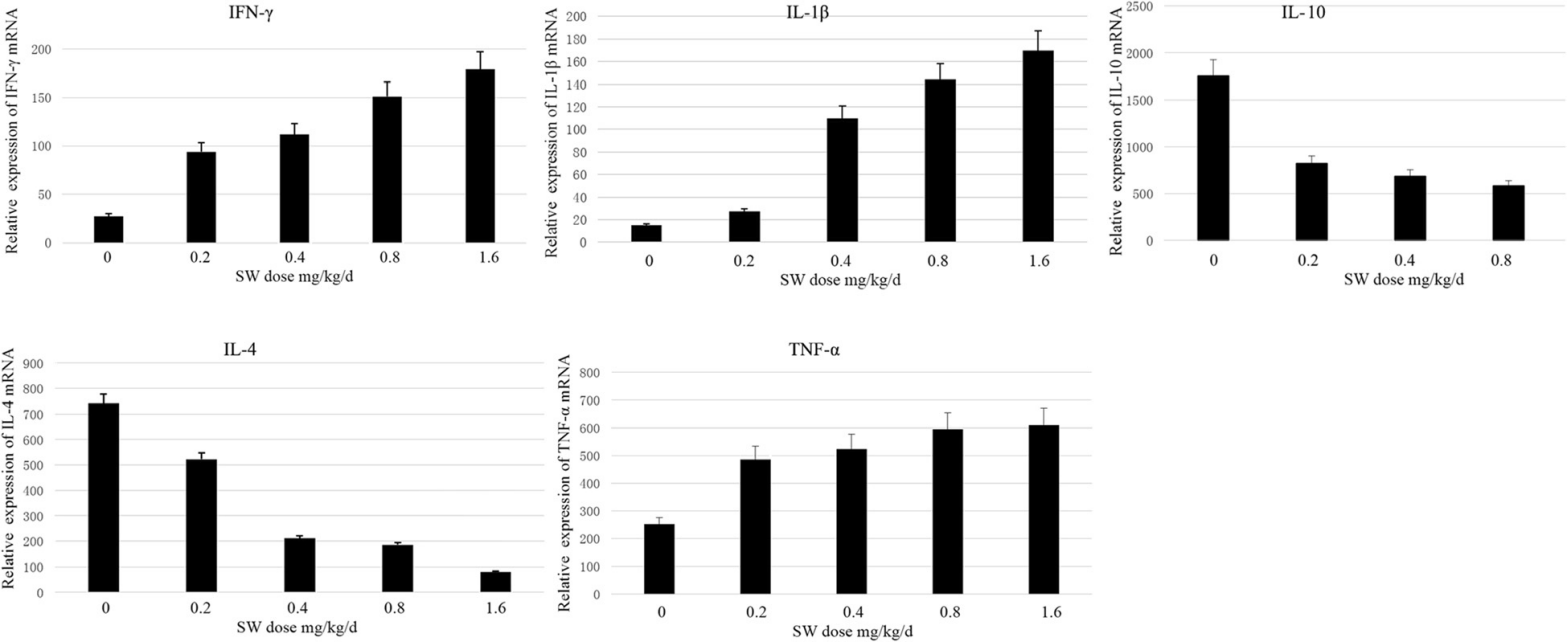
The expression of genes related to the Th1/Th2 cytokines. The relative expression level was measured by qRT-PCR. β-actin was used as an internal control. Each reaction was repeated three times. And the data was expressed as the mean ± SD, relative to the control. *means *P* < 0.05 and **means *P* < 0.01 as the indication of the different groups exposed to swainsonine compared with the control group. n = 3 for each replicate; three replicates were conducted

To investigate the effects of swainsonine on IL-1β, IFN-γ, TNF-α, IL-4 and IL-10, the expression level of mRNA was measured. The transcriptional responses of several cytokine’ genes are shown in Fig. 3. The expression of IL-1β, IFN-γ and TNF-α gene was significantly up-regulated (*P* < 0.05) after swainsonine exposure comparable control group, but the expression of IL-4 and IL-10 was significantly down-regulated (*P* < 0.05).

### Swainsonine promotes activity of Th1 cells and suppresses Th2

**Fig. 4:**
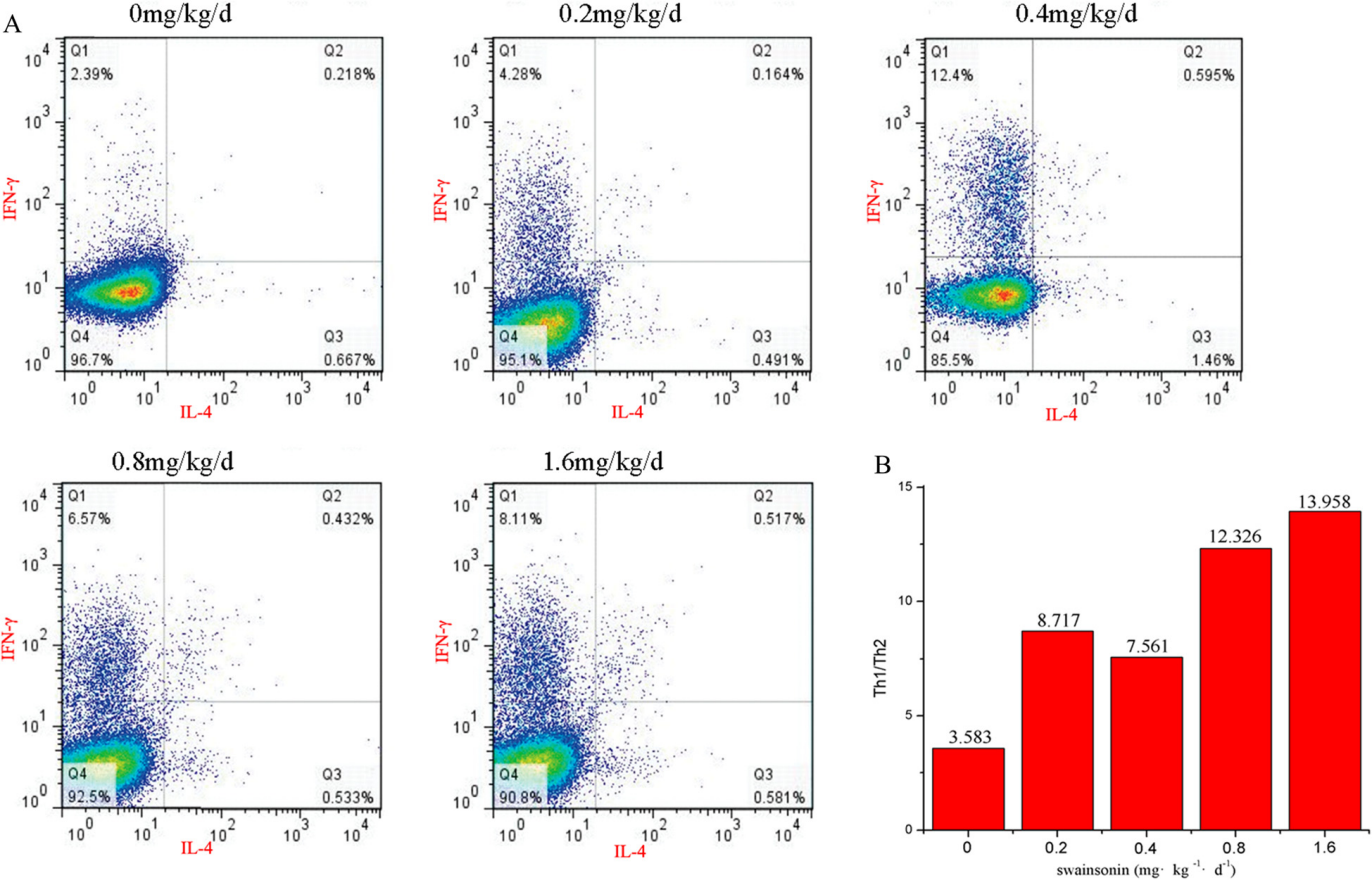
Flow cytometry of Th1/Th2 cytokines between four groups and control group (0 mg•kg^−1^•d^−1^) at baseline. As showed in the Fig. 3a and b, the Th1/Th2 balance of pregnant mice was imbalanced, respectively. **a** Were the intracellular cytokine expression of Th1 and Th2. The X-axis were the expression of IL-4 and the Y-axis were the expression of IFN-γ. **b** Were the Th1/Th2 of each group. Different dose of swainsonine can significantly effect Th1/Th2 balance. In the dose of 0.2 mg•kg^−1^•d^−1^ or higher dose, Th1 cell were promoted and Th2 were inhibited, especially in the dose of 1.6 mg•kg^−1^•d^−1^. Swainsonine-treaded mice showed Th1 dominant that is detrimental to pregnancy

Imbalance among the Th1 and Th2 cell immune responses plays an important role in unexplained habitual abortion (URSA) [25]. We investigated the Th1/Th2 subpopulations after T helper cell activation. The data showed that compared to control group, the proportion of Th1 (IFN-γ^+^) cell increased, while the proportions of Th2 (IL-4^+^) cell decreased markedly at the experiment groups (Fig. 4), resulting in the increased ratios of Th1/Th2 cells, which is not benefic to pregnancy.

## Discussion

In the past decades, swainsonine was a focus of research of the main toxic ingredient in locoweed and in the development of antineoplastic drugs. They showed that swainsonine can lead to animal’s miscarriage and stillbirth, probably involving corpus luteum and hormone excretion, and possibly a direct action to the fetus; nevertheless, there are no reports about immunotoxicity for reproduction [3, 8, 26, 27]. We demonstrate that dietary treatment of pregnant mice with different concentration of swainsonine for 13 days results in significant alterations in a number of parameters associated with the immunity of reproduction. We observed that different dose treatments lead to different levels of promotion of Th1 immune and inhibition of Th2 immune, as immunoenhancement was previously established using this regimen. Since it is well established that this regimen of exposure induces a measure of immunoenhancement [28–30]. We expected these studies would provide experimental data that could subsequently be used for related locoism analyses.

Previous studies have provided sufficient evidence that swainsonine can perform its cellular immune function by increasing organ index and the cell proliferation rate. The dose in our research was determined as 0 ~ 1.6 mg•kg^−1^•d^−1^ in accordance with previous studies and preliminary experiments that showed an immunoregulation effect but no abortion. The highest dose group of 3.2 mg•kg^−1^•d^−1^ was not done due to the occurrence of abortion or eutocia, despite a significant regulation of Th1/Th2 and other relevant cytokines [31, 32]. So the dose was determined to avoid abortion in the experiment. An easy way to determine whether a pregnant mouse has an abortion is by checking weight, because they normally increased 30 % during pregnancy.

In our research we found that IL-1 increased significantly in dose of 0.8 and 1.6 mg•kg^−1^•d^−1^ of swainsonine, but no difference were found in dose of 0.2 and 0.4 mg•kg^−1^•d^−1^. These findings are consistent with those of Hueza et al. [24], who reported that low dosages of *Ipomoea carnea*, a kind of swainsonine-containing plants, induced enhanced phagocytosis activity and hydrogen peroxide production by macrophages, as IL-1 is intensely produced by macrophages. On the other hand, Zhang et al. found that following exposure of adult KM mice to the dose of 0.2-0.8 mg•kg^−1^ by gavage over a period of 24 days, organ index and cellular counts in blood were increased and the proliferative activity of spleen lymphocytes were stimulated by swainsonine alone or combined with *ConA* and PHA-P, but cellular immune function would be depressed at the dose of 6.4 mg•kg^−1^ [33]. In addition, there are differences in sensitivity by species to swainsonine accumulation. TNF-α is one of the proinflammatory cytokines and can be classified as Th1-type cytokines in reproductive immunology. TNF-α and IFN-γ are the major effectors of phagocyte-mediated host defense, protective against intracellular pathogens. They induce miscarriage in mice, which can be reversed by inhibitors of the Th1 cytokines or by administering the anti-inflammatory IL-10 and Th2 cytokine IL-4. Previous studies have shown that TNF-α and IL-1β, proinflammatory cytokines ultimately result in the production of prostaglandins and MMPs, via NF-κB. This triggers a cascade of prolabour events including uterine contractility and fetal membrane rupture, and if this cascade is activated early in pregnancy, preterm labour can ensue. Moreover, term placentas exhibit comparatively higher levels of the IL-10 and Th2 cytokines, IL-4, compared with the preterm placentas.

At the same time, it is possible that swainsonine stimulates the Th1 arm of the cellular immune response and suppresses the Th2 arm of the humoral immune response [34]. Dennis, who disclosed a stable crystalline chloride or bromide salt of swainsonine and applied for a patent, insists that swainsonine have valuable pharmacological and immunomodulatory properties. Indeed, we consider that swainsonine is associated with a profound imbalance in Th1/Th2-type cytokines with excess type 1 and deficient type 2 responses. This imbalance was manifest in both inducing (IL-1 and IL-10) and effector (IFN-γ and IL-4) cytokines. IFN-γ and IL-4 are the most substantiated example of a pair of mutually counter-regulatory cytokines and represent the archetypal cytokines of the Th1/Th2-type paradigm. IL-10 is a product of various cell types including T-cells and monocytes and possesses a wide range of activities including suppression of Th1-type cytokine secretion. The significantly decreased quantity of IL-4^+^ to IFN-γ^+^ secreting cells ratio also demonstrates the development of an excessive type 1 and deficient type 2 immune response in the pregnant mice with the high dose exposure of swainsonine.

Th1 cells, which produce proinflammatory cytokines (IFN-γ), are involved in acute allograft rejection of transplanted tissues, and as the main immunotoxicity factor to fetus [14, 35]. Th2 cells, which produce cytokines, such as IL-4 and IL-10, are associated with allograft tolerance,and protected fetus from maternal Th1-cell attack [16]. During pregnancy, the balance of the Th1/Th2 paradigm skews to Th2 to protect the fetus from maternal Th1-cell attack [36]. Indeed, a predominant Th1-type immune response has been observed in pregnancy complications, such as recurrent spontaneous abortion (RSA) and eclampsia [37]. Therefore, adequate balance for Th1/Th2 immunity, slightly shifted to Th2-type immunity, may be suitable for the maintenance of pregnancy. Predominant Th1-type immunity may suppress the tolerance system by releasing pro-inflammatory cytokine or stimulating NK cell, neutrophilic cell , resulting in abortion [38].

The mechanism via which exposure of mice to swainsonine enhances Th1 cell activity and inhibit Th2 cell activity remains to be elucidated. However, one possibility is that this phenomenon may be a result of inhibition of Golgi α-mannosidase. Blocking the pathway at Golgi α-mannosidase II causes an accumulation of “hybrid-type” carbohydrate structures, which have terminal mannose residues. The exposed mannose residues are important features directly related to immune stimulation [39]. At the molecular level, it has been shown that certain cytokines, including IFN-γ, IL-1 and TNF-α, bind to carbohydrate structures terminating in mannose structures such as those which accumulate when Golgi mannosidase II is blocked [40]. As a result, blocking the pathway will indirectly affect the Th1/Th2 balance.

Swainsonine has valuable pharmacological properties and they provide antimicrobial, cancer suppressing effects, hemorestorative, chemoprotective, radioprotective, and immunomodulatory properties [41, 42]. It is confirmed that swainsonine-containing plant is effective against *Ptroteus vulgaris*, *Salmonella typhimurium* bacteria and *Aspergillus niger*, *Penicillium digitatum*, *Botrytis cinerea*, *Rhizopus arrhizus*, *Aspergillus flavus*, *Chaetomium brasiliense* and *Rhizoctonia solani* [42, 43]. Swainsonine induced macrophage tumoricidal activity and acted as an important immune effector involved in the suppression of tumor growth and metastasis in vital organs such as the lung, oesophagus [44, 45], liver and spleen [46]. We found that the intake of swainsonine or some swainsonine-containing plants as traditional medicine may pose a threat to the pregnancy. Therefore, the application of swainsonine as either drug or supplementary food should be reconsidered.

One standard procedure for assessing the reproduction immunological status is to determine the Th1/Th2 immunization [47]. We observed significant changes in this parameter in the pregnant Balb/c mice with the regimen of exposure. However, recent developments in studies of the implantation process and in the emergence of the uterine vascular bed and its control by natural killer cells and cytokines suggest that one must go beyond this hitherto useful scheme [48].

## Conclusion

The present findings demonstrate for the first time that at least certain swainsonine can exert adverse effect on the reproduction immunity, an important factor of pregnancy for all animals. Together, these findings provide strong support on our conclusion that at the dose of 0.4 ~ 1.6 mg•kg^−1^•d^−1^ swainsonine in the diet over a period of 13 days effect the Th1/Th2 paradigm of pregnant female Balb/c mice and is harmful to pregnancy. Determination of Th1/Th2 ratio, cytokine expression and mRNA level of IL-1, IL-6, IL-10, IFN-γ, TNF-α genes in pregnant mice exposed by swainsonine and reproduction immunology. The study may provide new aspects of that reproductive immunology that could be effected by swainsonine and its negative effect to animals in the pregnant period.

## Methods

### Animals and swainsonine exposures

10-week-old Balb/c female mice were obtained from Vital River Company. Thirty-six pregnant mice were divided equally into 6 groups and 6 mice for each group: one control group and five experimental groups. Swainsonine was provided by the laboratory of biotoxin and molecular toxicology of Northwest Agriculture and Forestry University in China. Pregnant mice in the experimental group were treated with 0.2, 0.4, 0.8, 1.6, 3.2 mg•kg^−1^•d^−1^ swainsonine by gavage daily for 13 days from 8th to 20th day after pregnancy through administration by orally, and pregnant mice in control group were feeding equal volume of water. All animals received food and water ad-libitum and were maintained under controlled temperature (22-25 °C), humidity (50 %-65 %) and lighting (12/12 light/dark cycle) conditions. Animals were treated humanely and with regard for alleviation of suffering. All experimental procedures with mice and animal care used in the present study had been given prior approval by the recommendations in the Guide for Sichuan Agricultural University Animal Care and Use Committee, Sichuan Agricultural University, Sichuan, China under permit no. DKY-B20100805. The field studies did not involve endangered or protected species. All surgery was performed under ether anesthesia, and all other efforts which were made to minimize suffering.

### Pregnancy situation

Pregnancy situation was observed every day, including appetite, drinking, faeces, and body weight. We considered that if the body weight lost a lot suddenly (more than 15 %), it was regarded that abortion has been occurred. Aborted mice would be abandoned. If the body weight gain was below expected, especially in the late pregnancy, we regarded that abortion occurred or the mice failed to get pregnancy.

### Histopathological slide

After 13 days of intoxication, mice were euthanized by cervical dislocation. Then corpus luteum samples were fixed in 4 % paraformaldehyde solution for 24 h. Specimens were embedded in paraffin, sectioned at 5-μm thicknesses, and stained with haematoxylin and eosin (HE), periodic acid-Schiff (PAS) for carbohydrates and Kluver-Barrera (KB), to detect cell vacuolation in corpus lutein regions by microscope (ix 70, Olympus).

### Flow cytometry analysis of Th1/Th2

To observe the Th1/Th2 paradigm in the PBMC of pregnancy mice, the heparinized peripheral blood was collected surgically before the mice were sacrificed. Peripheral blood mononuclear cells (PBMC) were isolated from heparinized peripheral blood diluted two fold with Dulbecco’s phosphate-buffered saline (PBS) using a density gradient (TBD. China) with centrifugation at 350 × g for 10 min. Isolated PBMC suspension was washed twice with PBS. These PBMC were resuspended in DMEM (Gibco. USA) with 10 % fetal bovine serum (Gibco, USA) at 1 × 10^6^/ml. Cells were cultured with PMA (25 ng/ml, Beyotime), Ionomycin (1 μg/ml, Beyotime) and Monensin (1.71 μg/ml, Beyotime) for 5 h at 37 °C and 5 % CO_2_. Upon harvest, cells were washed twice in PBS, then dealt with Fix&Perm kit (Beijing 4A Biotech Co., Ltd) according to the manufacturer’s instructions in 5 ml sterile tubes. After centrifugation, cells were divided equally, followed by incubating with 5 μl neutralizing antibodies CD4-PerCP-Cy5.5(30 μg/ml, 130-102-398; Miltenyi Biotec), anti-IL-4-APC (30 μg/ml, 130-102-398; Miltenyi Biotec) and anti- IFN-γ-PE (30 μg/ml, 130-102-388; Miltenyi Biotec) for 20 min in the dark at room temperature. After being washed in PBS, the cells were detected by a flow cytometer (Beckman Colter, USA) and the data analysis was performed using FlowJo software (version 7.6.2).

### Cytokine enzyme-linked immunosorbent assay (ELISA)

Blood was obtained by retro-orbital bleeding and the serum was collected by spinning at 2000 × g for 10 min after 60 min of clotting at room temperature. IL-1, IL-4, IL-10, IFN-γ and TNF-α were analyzed using ELISA kit (Shanghai ELISA, China ) according to the manufacturer’s protocols.

### Real-time PCR

PBMC were separated after the mice were euthanized, and RNA was extracted using TRIzol (Invitrogen, USA). RNA was quantitated using a spectrophotometer, and the same amount of RNA from each sample was used to make cDNA and was used as a template for real-time PCR. n = 3 for each replicate; three replicates were conducted. Each experimental time primer pairs were dropped on separate MicroAmp optical 96-well reaction plates using the 7500 Fast Real-Time PCR System (Applied Biosystems, USA). To determine the expression ratios of cytokine genes between cells induced to differentiate for various dose points, all values were normalized to β-actin according to equation:ΔCt = (Ct_baseline_ - Ct_trmt_)_YFG_ -(Ct_baseline_ - Ct_trmt_)_β-actin_, then the ratio between treatment and baseline was calculated as 2^|ΔCt|^. The sign of the value for indicates either up or down-regulation. The primer sequences used were: for β-actin, 5′- TGACCGAGCGTGGCTACA-3′ and 5′- TCTCTTTGATGTCACG CACGAT -3′, for IL-1β,5′- CAACCAACAAGTGATATT CTCCATG-3′, for IL-10, 5′-CTATGCTGCCTGCTCTTACTG-′3, 5′- AACCCAAGTAACCCTTAAAGTC-3′, for TNF-α,5′- ATCCGCGACGTGGAACTG -3′, 5′- ACCGCCTGGAGTTCTGGAA -3′. For IFN-γ and IL-4 TaqMan analysis, the murine IFN-γ and IL-4 analysis kit form Applied Biosystems was used.

### Statistical analysis

All experiments were repeated at least three times, with representative results shown. The data were expressed as mean ± standard deviation (SD). Statistical analysis was performed using SPSS software (Version 19 for Windows, SPSS, Italy SRL, Bologna, Italy). Statistical differences were determined using ANOVA followed by Dunnett’s multiple comparison test, the Tukey–Kramer multiple comparisons test or unpaired *t* test. The effects were considered significant if *P* ≤ 0.05.
